# Morphological Characteristics and Extracellular Matrix Abnormalities in Astrocytes Derived From iPSCs of Children With Alexander Disease

**DOI:** 10.1111/cns.70240

**Published:** 2025-01-27

**Authors:** Huan Yi, Jie Zhang, Kai Gao, Wei Yan, Hongyuan Chu, Junjiao Zhang, Fan Zhang, Yuwu Jiang, Jingmin Wang, Ye Wu

**Affiliations:** ^1^ Children's Medical Center, Department of Pediatric Neurology Peking University First Hospital Beijing China; ^2^ Beijing Key Laboratory of Molecular Diagnosis and Study on Pediatric Genetic Diseases Beijing China

**Keywords:** Alexander disease, extracellular matrix, GFAP, induced pluripotent stem cells, Rosenthal fibers

## Abstract

**Aims:**

Alexander disease (AxD) is a leukodystrophy caused by mutations in the astrocytic filament gene *GFAP*. There are currently no effective treatments for AxD. Previous studies have rarely established AxD models with the patient's original GFAP mutations. In this study, we aimed to explore the morphological and transcriptomic characteristics of GFAP‐mutant astrocytes via induced pluripotent stem cell (iPSC) models of AxD.

**Methods:**

Fibroblasts from three AxD children were reprogrammed into iPSCs. Wild‐type (WT) and AxD‐iPSCs were differentiated into astrocytes. We compared the morphological and transcriptomic differences between WT‐ and AxD iPSC–derived astrocytes.

**Results:**

Astrocytes induced from AxD‐derived iPSCs exhibited the Rosenthal fibers (RFs), the main pathological phenotype of AxD. Compared with WT astrocytes, AxD astrocytes had shorter processes, more branches, and larger cell bodies. Transcriptomic analysis revealed that extracellular matrix (ECM) components, particularly chondroitin sulfate proteoglycans (CSPGs), were upregulated, and ECM‐degrading enzymes were generally downregulated. These changes may lead to abnormalities in neurons and myelination.

**Conclusions:**

We explored the morphological characteristics of AxD astrocytes via iPSC models and revealed the ECM, previously unexplored for AxD, may be an important new pathogenic mechanism of this disease.

## Graphical abstract

Astrocytes derived from Alexander disease (AxD) patient iPSCs simulated the pathological phenotypes of AxD and exhibited abnormal morphology. Mutant astrocytes showed abnormal expression of extracellular matrix components, which may further lead to myelin and neuronal abnormalities.

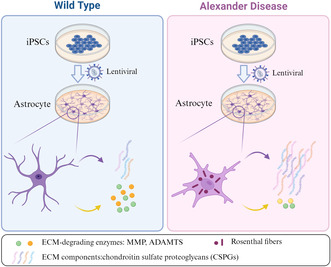

## 1 Introduction

Alexander disease (AxD) is an autosomal dominant leukodystrophy caused by mutations in the gene encoding glial fibrillary acidic protein (GFAP) [1, 2], which is an intermediate filament‐III protein uniquely found in astrocytes in the central nervous system (CNS) [3]. Thus, AxD is considered to be a primary disease of astrocytes [4]. Under physiological conditions, GFAP polymerizes into intermediate filaments, a part of the cytoskeleton that is responsible for maintaining astrocyte morphology [3]. However, in AxD, mutant GFAP fails to polymerize properly and tends to aggregate [1]. The aggregated GFAP, along with the subsequent recruitment of ubiquitin, heat shock proteins, vimentin, apoptotic proteins, and other proteins, forms dense intracellular inclusions known as Rosenthal fibers (RFs) [5], which are the hallmark pathology of AxD. RF accumulation leads to astrocyte dysfunction, further resulting in CNS hypomyelination and neurotoxicity [6, 7], which is manifested pathologically as disorganization or absence of myelin, as well as axonal destruction [2, 8, 9, 10].

Most AxD patients present before 4 years of age, with clinical manifestations including seizures, macrocephaly, delayed intellectual and motor development, encephalopathy, and episodic neurological deterioration. The average survival time after onset is only 14 years [11, 12]. Therefore, identifying the key mechanisms and developing new therapeutic approaches are urgently needed.

Human induced pluripotent stem cells (iPSCs) retain the original mutations from patients and can be differentiated into any cell type of interest, making them an ideal model for studying genetic diseases and providing a valuable platform for further research into disease mechanisms and drug screening [13]. In this study, we generated iPSC lines from three AxD patients with different mutation sites. We subsequently differentiated three AxD‐iPSC lines and three WT iPSC lines into astrocytes and compared their pathological phenotypes and morphological differences. We summarized the unique morphological characteristics of astrocytes derived from AxD‐iPSCs. By comparing transcriptomic differences, we focused, for the first time, on the molecular mechanisms of AxD in the extracellular matrix (ECM), which plays an important role in many CNS diseases.

## 2 Methods

### 2.1 Patients and Sample Collection

Skins were obtained from three patients with AxD, and the samples were named AxD1, AxD2, and AxD3. The inclusion criteria were as follows: (1) onset before 18 years of age; (2) radiological and clinical features consistent with AxD; and (3) genetic testing revealing likely pathogenic or pathogenic variants in the *GFAP*. The study was approved by the Biomedical Ethics Committee of Peking University First Hospital, and informed consent was obtained from the parents. Low‐passage skin fibroblasts are then used for subsequent reprogramming into iPSCs.

### 2.2 Generation of Human iPSCs

We used the ReproRNA‐OKSGM kit (STEMCELL Technologies, 05930), a single‐stranded RNA replicon vector, for reprogramming. Skin fibroblasts were seeded at 1 × 10^5^ cells/cm^2^ in Matrigel (Corning, 354,277)‐coated 6‐well plates, and the next day, they were transfected with a vector containing five reprogramming factors (OCT4, KLF‐4, SOX2, GLIS1, and c‐MYC). After 1 week of culture in growth medium (advanced DMEM supplemented with 10% FBS, 2 mM L‐glutamine and 175 ng/mL recombinant B18R protein), the medium was changed to ReproTeSR (STEMCELL Technologies, 05926) for another week. By Day 15, iPS cell colonies had become visible. The colonies were picked and cultured in mTeSR (STEMCELL Technologies, 85850), and iPSCs from passages 20 to 40 were used for experiments.

### 2.3 Differentiation of Astrocytes From Human iPSCs

We used lentiviruses overexpressing SOX9 and NFIB to induce the differentiation of iPSCs into astrocytes ([14]; Figure 2B). In addition to the three AxD‐iPSC lines, three WT iPSC lines (WT‐B1, WT‐F1, and WT‐U2) were also used as healthy controls in the experiments. All WT‐iPSCs were provided by Beijing Cellapy Biotechnology Co. Ltd. Before differentiation, all iPSCs were dissociated into single cells with accutase (Gibco, A1110501) and seeded into GFR Matrigel (Corning, 356230)‐coated 6‐well plates at a density of 2 × 10^5^ cells/well in mTeSR containing 10 μM Y27632 (STEMCELL Technologies, 72304). The next day (Day 0), the cells were infected with lentivirus, MOI = 10. On Days 1 and 2, the medium was changed to expansion medium (DMEM/F12, 10% FBS, 1% N2 supplement, and 1% GlutaMAX), and 1.25 μg/mL puromycin (Leagene, CA0069) and 3 μg/mL blasticidin (Beyotime, ST018) were also added to the medium for negative selection. From Day 3 to Day 6, the expansion medium was gradually replaced with FGF medium (neurobasal medium supplemented with 2% B27, 1% nonessential amino acids, 1% GlutaMAX, 10% FBS, 5 ng/mL CNTF, 10 ng/mL FGF, and 10 ng/mL BMP4). On Day 7, the cells were dissociated with accutase, replated, and maintained in FGF medium. Beginning on Day 10, the cells were maintained with maturation medium (DMEM/F12: Neurobasal = 1:1, 1% N2, 1% sodium pyruvate, 1% GlutaMAX, 5 μg/mL N‐acetyl‐cysteine, 10 ng/mL CNTF, 10 ng/mL BMP4, 5 ng/mL heparin‐binding EGF‐like growth factor, and 1 mM dbcAMP), and the medium was changed every 2 days. On Day 21, most of the cells differentiated into astrocytes, which were used for further experiments.

### 2.4 Immunofluorescence Study

Immunofluorescence staining was performed as previously described [15]. Briefly, the cells were incubated at room temperature for 10 min in 4% paraformaldehyde (pH 7.4) and then washed with PBS three times. The cells were then permeabilized for 10 min with 0.3% Triton X‐100 and blocked for 60 min at room temperature in 5% bovine serum albumin (BSA). The primary antibodies were incubated overnight at 4°C, and the fluorescent dye‐conjugated secondary antibodies were incubated for 1 h at room temperature. Hoechst (Thermo Fisher Scientific) was used for nuclear staining. Images were captured with a microscope (Fv10‐ASW, Olympus). The following primary antibodies were used: Oct4 (Abcam, ab181557, 1:250), Nanog (Abcam, ab109250, 1:250), Sox17 (Abcam, ab224637, 1:1000), Brachyury (Abcam, ab209665, 1:1000), Nestin (CST, 33475, 1:1600), Sox2 (CST, D9B8N, 1:400), GFAP (CST, 3670, 1:400), S100β (CST, 90393, 1:800), Vimentin (CST, 5741, 1:100), and CRYAB (Santa Cruz Biotechnology, sc‐137,143, 1:500).

### 2.5 Transmission Electron Microscopy

Briefly, iPSC‐derived astrocytes were collected in centrifuge tubes, washed three times with PBS, supplemented with 2.5% glutaraldehyde fixative, fixed at 4°C overnight, fixed with 1% osmium tetroxide for 90 min, dehydrated with ethanol, and embedded in Epon. The cells were cut into ultrathin sections with an ultramicrotome, mounted on grids, and stained with uranyl acetate and lead citrate. Finally, the slices were observed and analyzed via transmission electron microscopy.

### 2.6 Astrocyte Morphological Analysis

We used consistent parameters for fluorescence microscopy imaging to compare astrocytes across different groups. For each group of samples, we selected high‐magnification fields of astrocytes from three independent experimental replicates for statistical analysis. To analyze the morphological characteristics of astrocytes, we used ImageJ (imagej.nih.gov/ij/) to trace the astrocyte processes and performed Sholl analysis [16]. The distribution of astrocyte processes was analyzed by counting the intersections between the processes and Sholl shells. In addition, the process length, number of branches, branch length, branch distance from the center, cell body size, and cell body fluorescence intensity were also recorded. The data are expressed as the means ± SEMs, and *p* values < 0.05 were considered significant.

### 2.7 Transcriptome Analysis

After 21 days of induction, we collected RNA from astrocytes derived from three WT iPSCs and three AxD‐iPSCs, and RNA was extracted for RNA‐seq via TRIzol (Invitrogen, 15596018). Two micrograms of RNA per sample were used to generate cDNA libraries with the mRNA‐seq Lib Prep Kit for Illumina (ABclonal, RK20302). The library quality was controlled sequentially via a Qubit 2.0 fluorometer, an Agilent 2100 Bioanalyzer, and qPCR. High‐throughput sequencing of the libraries was subsequently performed on the DNBSEQ‐T7 platform. The raw data were processed for quality control via Trimmomatic (v0.36) software, which involves removing adapters and low‐quality reads. The reference genome alignment and analysis were conducted sequentially via HISAT2 (v2.2.1), GATK (v3.5), SplAdder (v2.4.2), and STAR‐Fusion (v1.10.1). Differentially expressed genes (DEGs) were identified via EBSeq (v1.26.0). In this study, we defined DEGs as those whose *p* value was < 0.05 and whose absolute value of log_2_(fold change) was > 1. GO and KEGG enrichment analyses of the DEG sets were executed via the Database for Annotation, Visualization, and Integrated Discovery (DAVID) v6.8. Benjamini–Hochberg procedure was performed to control the false discovery rate (FDR). GO terms and KEGG pathways with adjusted *p* values < 0.05 were considered significantly enriched with DEGs.

### 2.8 Statistics

Statistical analysis was performed via SPSS v25.0 and GraphPad Prism v8.0. The normal distribution of the data was checked via the Kolmogorov–Smirnov test, and the homogeneity of variance was checked via the *F* test. For normally distributed data, statistical significance was determined by one‐way ANOVA; otherwise, Kruskal–Wallis test were performed. All results were obtained from at least three independent repeat experiments. The data are expressed as the means ± SEMs, and *p* values < 0.05 were considered significant.

## 3 Results

### 3.1 Genotype and Clinical Characteristics of Three AxD Patients

AxD‐iPSCs were obtained from three patients with different heterozygous de novo *GFAP* mutations: p.R88C (AxD1), p.R239C (AxD2), and p.N77S (AxD3) (Table 1); among these, p.R239C is a hotspot mutation. All three patients had disease onset before 2 years of age. Two of the patients (AxD1 and AxD2) presented with motor delay as their onset symptom, which is the most common symptom of AxD [17]. Another patient (AxD3) presented with recurrent epileptic seizures as the onset symptom and subsequently developed motor delay. The disease in the AxD3 patient progressed rapidly, with recurrent episodes of status epilepticus that were unresponsive to multiple antiepileptic medications, ultimately leading to death at 21 months of age. The AxD1 patient was previously able to walk independently but later experienced motor regression and could only walk with support. This patient experienced seizures at 150 months of age. The AxD2 patient were never able to walk independently, experienced their first seizure at 18 months of age.

**TABLE 1:** Clinical information of AxD patients in this study.

Categories	AxD1	AxD2	AxD3
GFAP genotype (NM_002055)	c.262C>T (de novo)	c.715C>T (de novo)	c.230A>G (de novo)
Amino acid change	p.R88C	p.R239C	p.N77S
Sex	F	M	F
Onset age	3 months old	18 months old	3 months old
Age of skin sample collection	144 months old	30 months old	4 months old
Onset symptom	Motor delays	Motor delays	Epileptic seizures
Other symptoms	Motor regression, speech and language delays, epileptic seizures, hypertonia, and scoliosis	Epileptic seizures, status epilepticus, and hypertonia	Status epilepticus, motor delays, and hypotonia
Exacerbation by stress	Yes	No	No
Age at last follow‐up	165 months old	36 months old	21 months old
Last follow‐up status	Walk with support, swallowing difficulties, and seizure‐free for nearly 5 years on oral LEV	Walk with support, seizure‐free for nearly 1 year on oral LEV, and normal cognitive development	Deceased
GMFCS level at last follow‐up	3	2	6

Brain MRI (Figure 2B) of three AxD patients revealed bilateral white matter high signals on T2 and T2 FLAIR and low signals on T1. On the MR image of AxD1, the abnormal signal was located mainly in the bilateral periventricular white matter, whereas on the MR images of AxD2 and AxD3, it was located mainly in the bilateral subcortical white matter. Additionally, MRI of AxD2 and AxD3 also revealed swelling in the basal ganglia.

### 3.2 Generation of iPSCs

**FIGURE 1:**
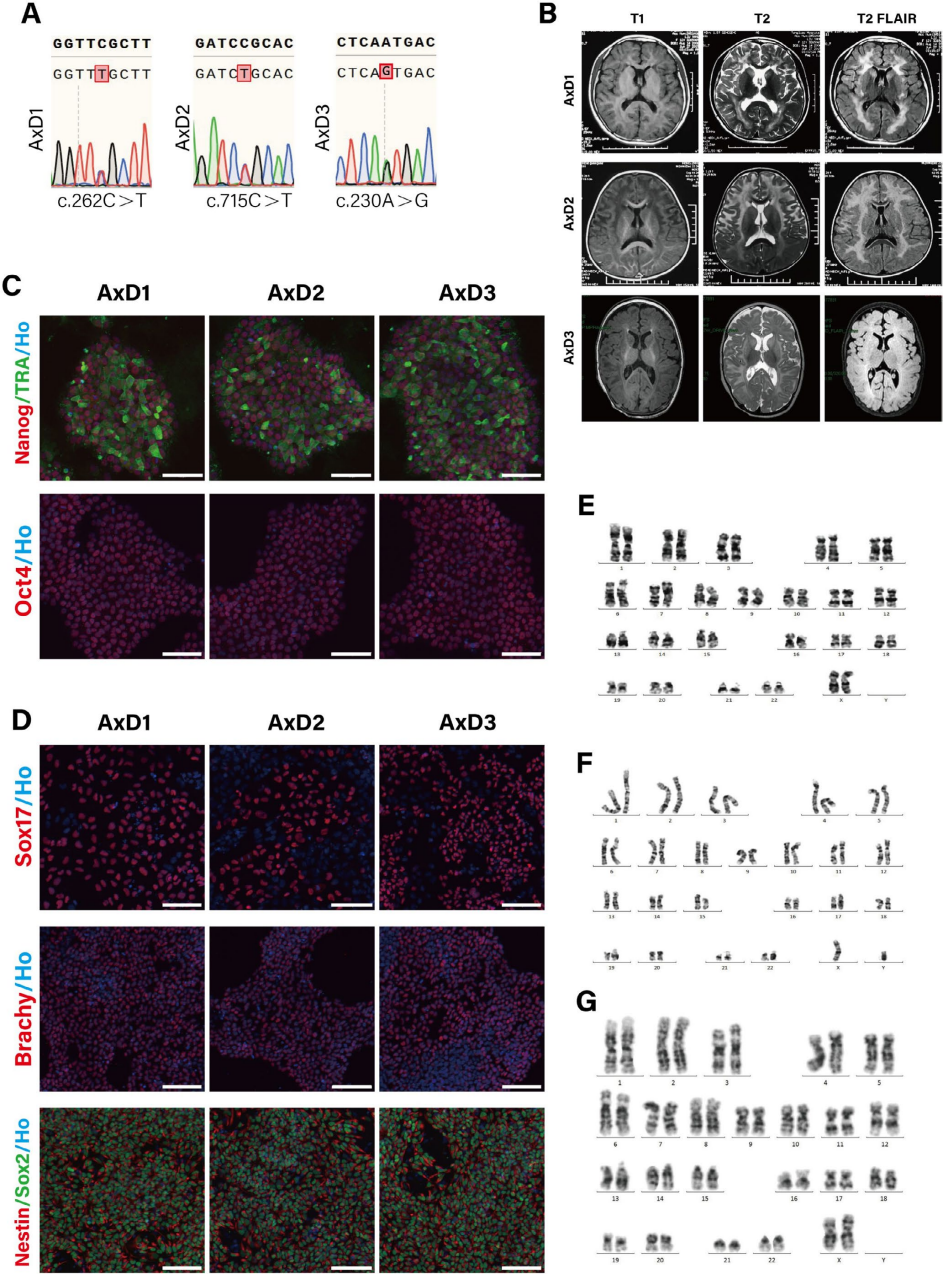
AxD patient‐derived iPSCs and identification. (A) *GFAP* mutations in AxD‐iPSC lines shown by Sanger generation sequencing. (B) Brain MRI of three AxD patients: MRI of AxD1 patient (154 months old) showed bilateral periventricular white matter, and the subcortical white matter of the frontal lobes had symmetrical slightly low signals on T1 and high signals on T2 and T2 FLAIR. MRI of AxD2 patient (22 months old) showed extensive low signals on T1 and high signals on T2 and T2 FLAIR in the bilateral subcortical white matter, especially prominent in the frontal regions, bilateral caudate nucleus and putamen swelling. MRI of AxD3 patient (4 months old) showed diffuse low signals on T1 and high signals on T2 and T2 FLAIR in both the subcortical white matter and basal ganglia regions and swelling of the basal ganglia and thalamus. (C) Immunofluorescence staining of pluripotent markers, OCT4, Nanog, and TRA‐1‐60. Scale bar, 100 μm. (D) Immunofluorescence staining of three germ layer markers: Endoderm (SOX17), mesoderm (Brachyury), and ectoderm (Nestin, Sox2). Scale bar, 100 μm. (E–G) Karyotype analysis of AxD1‐iPSCs, AxD2‐iPSCs, and AxD3‐iPSCs: 46, XX(E), 46, XY(F), 46, XX(G). Ho, Hoechst; Brachy, Brachyury.

Three AxD‐iPSC lines were derived from skin fibroblasts reprogrammed with a single‐stranded RNA replicon vector reprogramming kit. A mutation in AxD3 (c.230A>G) was established for the first time as an iPSC model. All the iPSCs expressed the pluripotency markers Oct4, NANOG and TRA1‐60 ([18]; Figure 1C). The in vitro differentiation of the three embryonic germ layers of iPSCs used in this research was also verified (Figure 1D). Differentiated cells expressed the ectoderm markers Nestin and Sox2, the mesoderm marker Brachyury and the endoderm marker SOX17 [19]. In addition, all the iPSCs displayed normal karyotypes (Figure 1E–G).

### 3.3 The Duration and Efficiency of the Induction of Mutant and WT iPSCs Into Astrocytes Were Comparable

**FIGURE 2:**
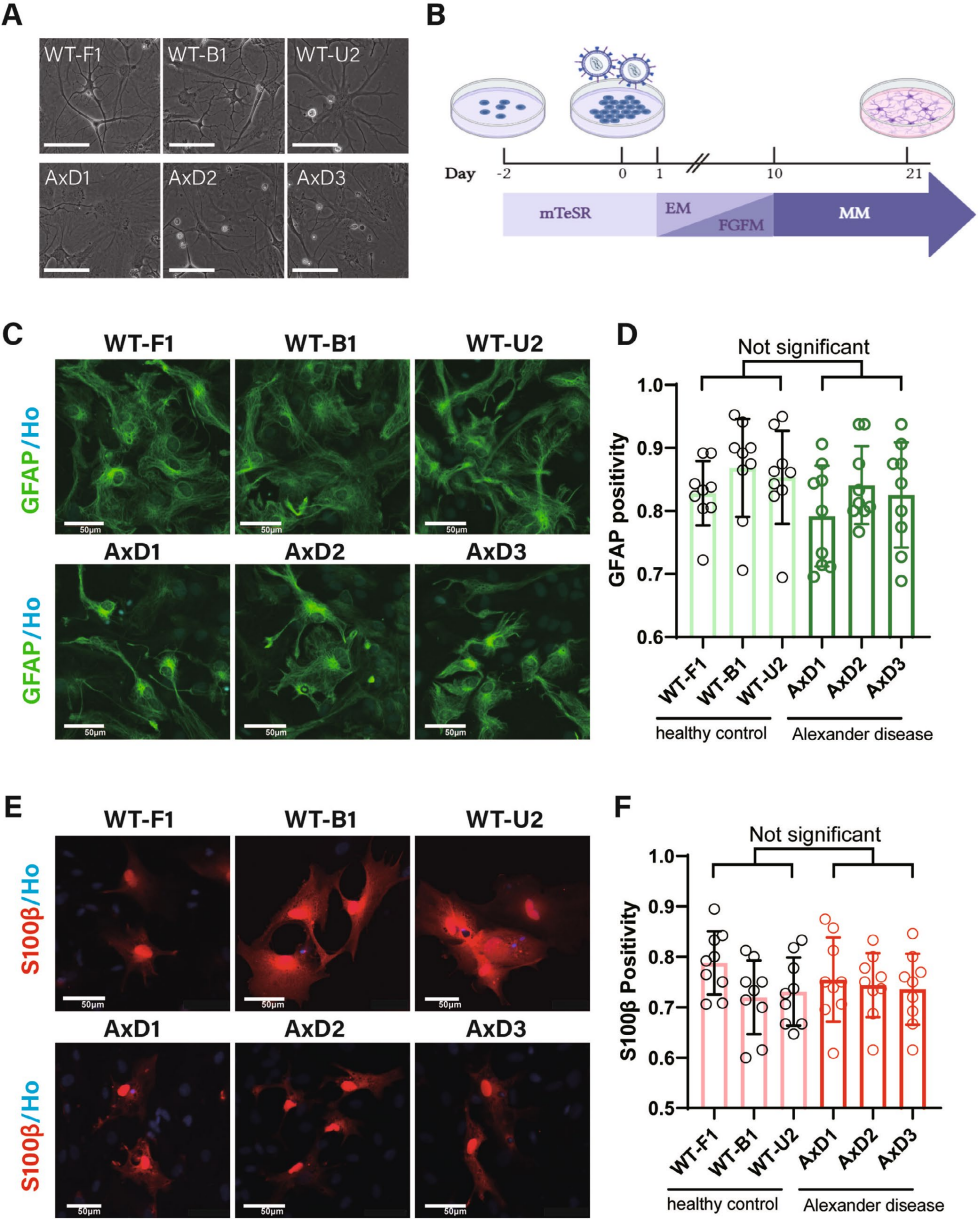
AxD‐iPSCs and WT‐iPSCs could differentiate into high‐purity astrocytes. (A) Astrocytes derived from both WT‐iPSCs and AxD‐iPSCs exhibited typical astrocytic morphology under a light microscope. (B) Schematic procedures for astrocyte differentiation. (C) Astrocytes derived from both WT‐iPSCs and AxD‐iPSCs expressed the astrocyte marker GFAP (green) and nuclear staining (Hoechst, blue); scale bars, 50 μm. (D) The percentage of GFAP‐positive astrocytes differentiated from each iPSC line was approximately 80%, with no significant difference. The data are presented as dot plots with means ± SDs; *n* = 9 in each group of three independent experiments; one‐way ANOVA. (E) Astrocytes derived from both WT‐iPSCs and AxD‐iPSCs expressed the astrocyte marker S100β (red); scale bars, 50 μm. (F) The S100β positivity rate of astrocytes differentiated from each iPSC line was approximately 80%, with no significant difference. The data represent as dot plots with means ± SDs, *n* = 9 in each group of three independent experiments, one‐way ANOVA. EM, Expansion Medium; FGFM, FGF Medium; MM, Maturation Medium; Ho, Hoechst.

To explore the mechanism of AxD and identify abnormalities in astrocytes caused by GFAP mutation, we differentiated three AxD‐iPSCs and three WT iPSCs (WT‐F1, WT‐B1, and WT‐U2) into astrocytes. The differentiation protocol involves rapid induction of iPSCs into astrocytes via lentivirus‐mediated overexpression of the transcription factors SOX9 and NFIB. We generated mature astrocytes after only 21 days of induction. This approach is much shorter than the traditional induction method, which usually takes several months to half a year [20, 21]. We compared the rapid induction protocol used in this study and traditional induction protocols (Figure S2). Both protocols are similar in their differentiation processes and are capable of inducing iPSCs to differentiate into astrocytes. Both AxD and WT iPSC‐derived astrocytes presented typical astrocyte morphology (Figure 2A) and abundantly expressed the mature astrocyte markers GFAP and S100β (Figure 2C,E). We also confirmed astrocytic identity by immunofluorescence assessment of the expression of ALDH1L1 and EAAT2 (Figure S1). The percentage of GFAP‐ and S100β‐positive astrocytes among WT iPSC–derived astrocytes and AxD‐iPSC‐derived astrocytes was approximately 80%, with no significant difference.

### 3.4 RFs Aggregate in AxD Astrocytes

RFs are the hallmark pathology of AxD, and previous studies have suggested that RF is composed of GFAP and other intermediate filament proteins and heat shock proteins, such as vimentin (VIM) and aB‐crystallin (CRYAB) [5].

**FIGURE 3:**
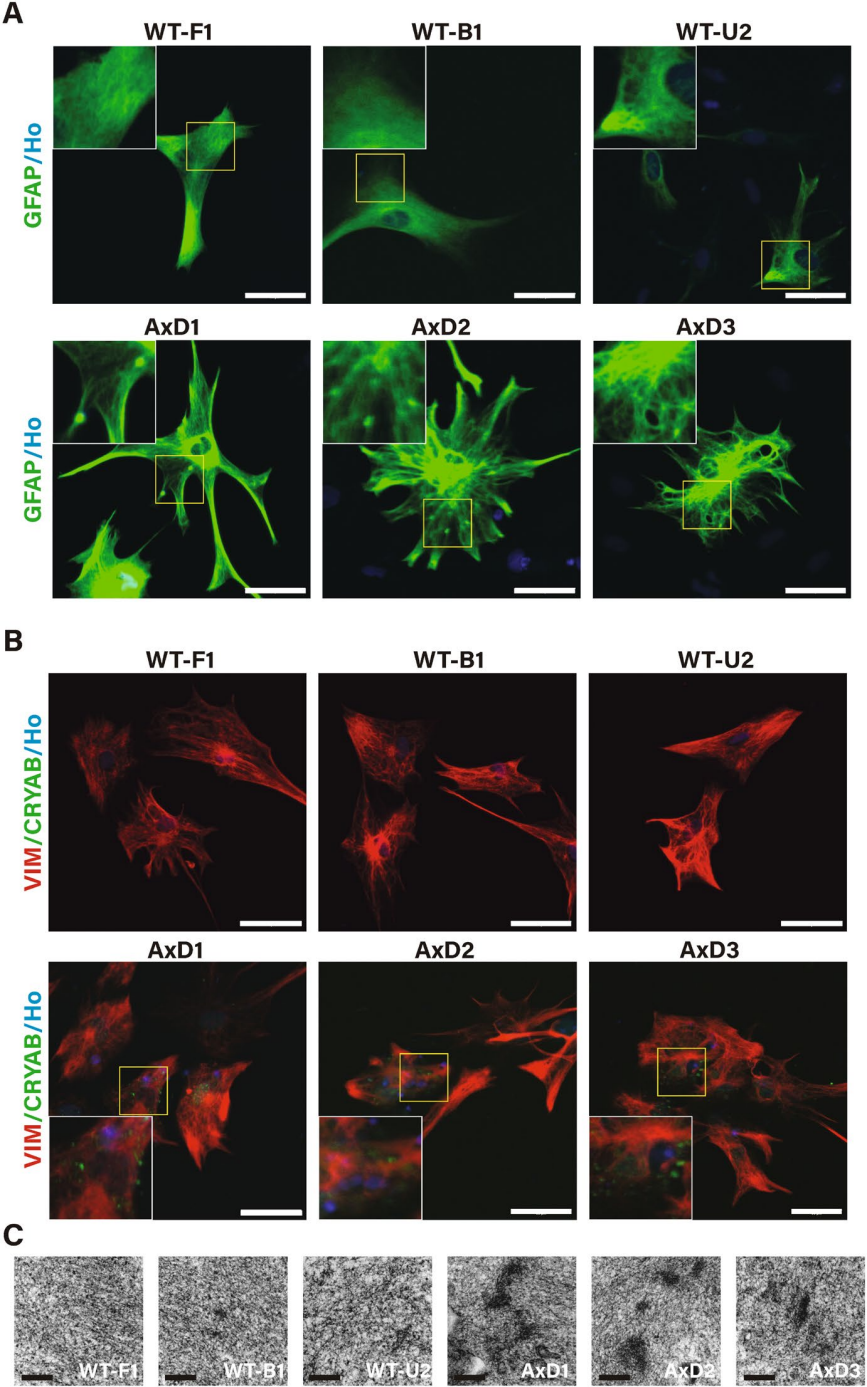
RFs were observed in AxD astrocytes. (A) In high‐magnification fields of GFAP (green) staining, GFAP‐positive aggregates were observed in three AxD astrocytes, whereas no GFAP aggregation was detected in the WT astrocytes. Scale bars, 50 μm. (B) In high‐magnification fields of RF marker staining, VIM (red) and CRYAB (green), VIM‐positive aggregates colocalized with CRYAB‐positive areas in AxD astrocytes, whereas in WT astrocytes, VIM aggregation and CRYAB expression were not detected. Scale bars, 50 μm. (C) Transmission electron microscopy of AxD astrocytes revealed electron‐dense deposits in the cytoplasm. Scale bars, 500 nm. VIM, Vimentin; Ho, Hoechst.

In our study, GFAP immunofluorescence staining revealed that intermediate filaments aggregated in AxD astrocytes (Figure 3A), and bundles of GFAP filaments resembled RFs in the brains of AxD patients [1, 6]. In contrast, WT‐iPSC‐derived astrocytes had fine intermediate filaments with a cytoskeleton. We also immunostained CRYAB and VIM which involved in the formation of RFs (Figure 3B). There was VIM aggregation in AxD astrocytes, which did not exist in WT astrocytes. CRYAB was observed in the cytoplasm of AxD astrocytes and colocalized with VIM aggregation, but it was not observed in WT astrocytes. Transmission electron microscopy also confirmed the RFs aggregation (Figure 3C). In AxD astrocytes, electron‐dense deposits on the intermediate filaments were observed, which confirmed that RFs originate from mutant GFAP, heat shock proteins, and other components deposited on intermediate filaments [22].

### 3.5 AxD Astrocytes Exhibited Unique Morphological Characteristics

**FIGURE 4:**
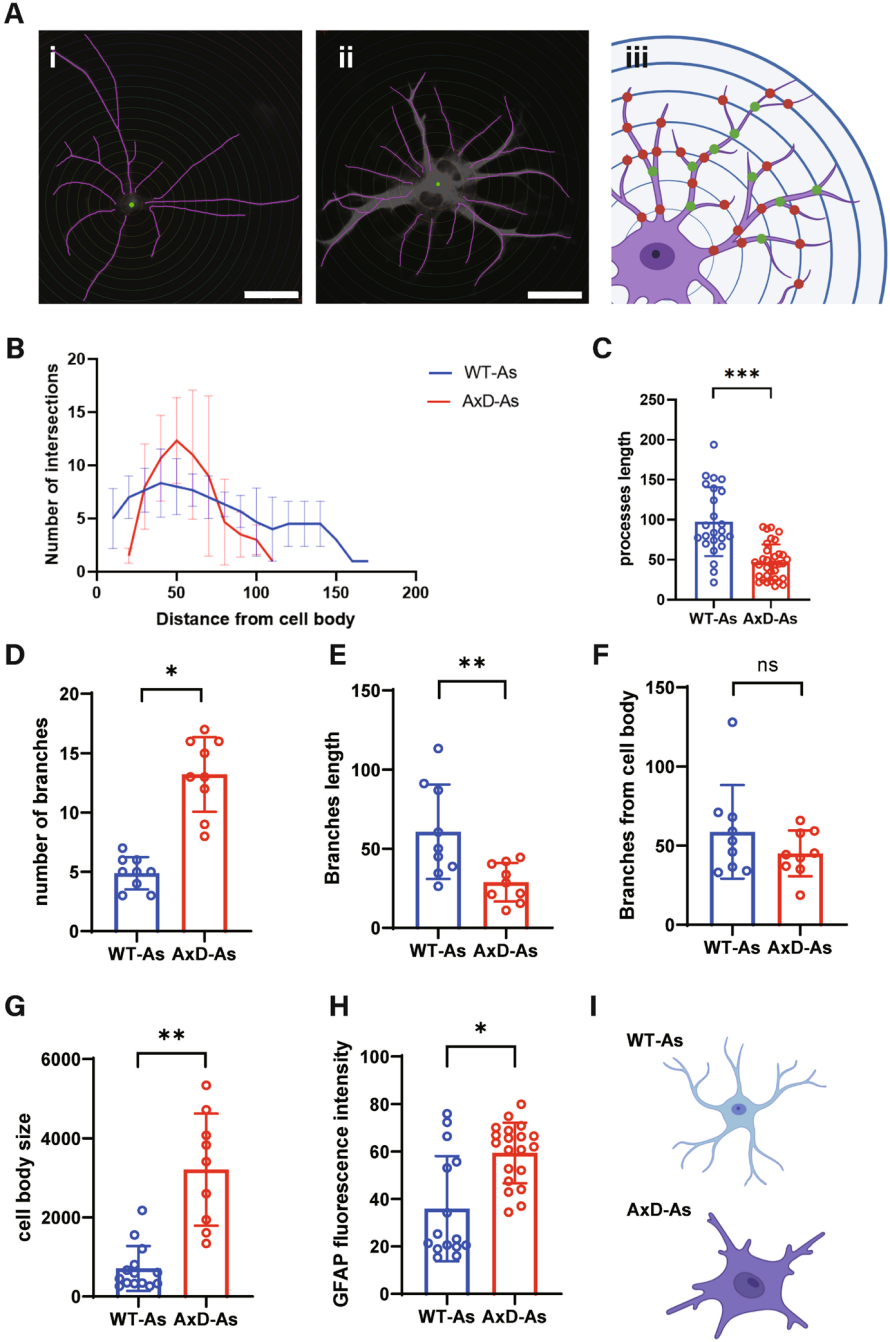
Sholl analysis of astrocyte process morphology. (A): Illustration of Sholl analysis. For astrocytes derived from WT‐iPSCs (Ai) and AxD‐iPSCs (Aii), tracing lines were drawn for the processes (purple lines in the images), and concentric circles (termed Sholl shells) were set 10 μm apart, with the cell body in the middle. The analysis involved counting intersections of concentric circles and processes (red points in Aiii) as well as branching points of the processes (green points in Aiii) to assess the morphological characteristics of the astrocytes. (B) Number of intersections between processes and concentric circles of different radii. The intersections of processes in AxD astrocytes are predominantly distributed approximately 50 μm from the center of the cell body, whereas in the WT group, the intersections are evenly distributed from 10 to 150 μm from the center. (C) The process length of WT astrocytes was longer than that of AxD astrocytes (*p* < 0.001). (D) Compared with WT astrocytes, AxD astrocytes presented more process branches (*p* < 0.05). (E) The branch length of AxD astrocytes was shorter than that of WT astrocytes (*p* < 0.01). (F) The average distance of branch points from the center in WT astrocytes was farther than that in AxD astrocytes, but the difference was not statistically significant. (G, H) Compared with WT astrocytes, AxD astrocytes had larger cell bodies (*p* < 0.01) and stronger GFAP fluorescence intensity (*p* < 0.05). (I) Morphological diagram of WT astrocytes (above) and AxD astrocytes (below) based on morphological analysis. Data represent as dot plots with mean ± SD, *n* = 9–35 in each group with three independent experiments, one‐way ANOVA (*p* = 0.000), ns: *p* > 0.05, *p < 0.05, **p < 0.01, ***p < 0.001, WT‐As, WT Astrocytes; AxD‐As, AxD Astrocytes.

Under physiological and pathological conditions, astrocytes are divided into different subtypes, including physiological states and a variety of reactive states [23], which have diverse sets of morphology and protein expression. In this study, we used Sholl analysis and ImageJ to explore the unique morphology of AxD astrocytes [24]. For the Sholl analysis, we drew a family of concentric circles centered on the center of the astrocyte cell body and recorded the intersections where the concentric circles intersected the cell process (Figure 4A), and analyzing the number of intersections and the distance from the center of the circle revealed the length and complexity of astrocyte processes.

The data indicated that intersections in the AxD‐astrocyte processes were distributed mainly within 50 μm, and the farthest region was 100 μm. In contrast, the intersections in WT astrocyte processes were evenly distributed from 10 to 150 μm away from the cell body (Figure 4B). This finding indicated that most WT astrocyte processes extended up to 150 μm from the cell body with few branches, whereas AxD‐astrocyte processes were generally shorter than 100 μm and exhibited abundant branching at approximately 50 μm. The subsequent statistical results also confirmed this finding.

When the average length of astrocyte primary processes was calculated, the process length of WT astrocytes was significantly greater than that of AxD astrocytes (Figure 4C). In addition to primary processes, branches are also important for the analysis of astrocyte morphology. According to the statistical results, compared with WT astrocytes, AxD astrocytes presented more branches (Figure 4D), the distance of branches starting point from cell body was shorter (Figure 4F), and the branches length was shorter (Figure 4E). In addition, AxD astrocytes had larger cell bodies (Figure 4G) and stronger fluorescence intensity of GFAP (Figure 4H). Based on the above morphological analysis, AxD astrocytes have larger bodies, overexpress GFAP, and have shorter but more complex and more branching processes (Figure 4I).

### 3.6 Transcriptomic Analysis Suggested That the Differentially Expressed Genes Were Enriched Mainly in the ECM

To explore the mechanism of AxD and identify the key molecules that lead to the pathology of AxD, we performed RNA sequencing (RNA‐seq) to analyze differentially expressed genes (DEGs) between WT astrocytes and AxD astrocytes.

**FIGURE 5:**
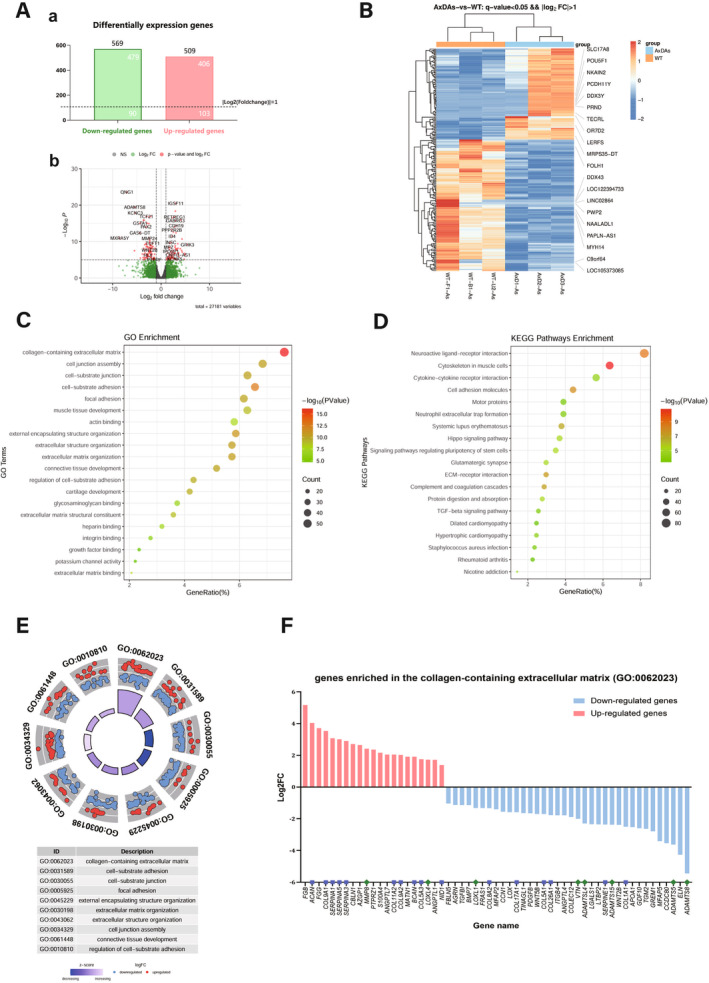
Transcriptome analysis reveals differences between AxD and WT astrocytes. (Aa) Number of DEGs between AxD and WT astrocytes; the numbers above the bar chart represent the total number of DEGs (569 genes downregulated, 509 genes upregulated). Genes above the dashed line have an absolute value of log_2_(fold change) > 1, whereas genes below the dashed line have an absolute value of log_2_(fold change) < 1. (Ab) Volcano map of gene expression changes in AxD astrocytes. The red dots represent genes with an absolute value of log_2_(fold change) > 1 and *p* < 0.05, the green dots represent genes with an absolute value of log_2_(fold change) < 1, and the gray dots represent genes with *p* < 0.05. (B) Heatmap of DEGs between WT astrocytes and AxD astrocytes. Each row represents a gene, and each column represents a sample. Gene expression levels are shown as normalized *Z*‐scores, with red indicating upregulation and blue indicating downregulation. (C, D) GO term and KEGG pathway enrichment of DEGs. Bubbles represent the most enriched GO (C) and KEGG (D) analysis terms. The size of the bubble indicates the number of enriched genes, whereas the color intensity of the bubble represents the P value. The vertical axis represents the name of the relevant terms, and the horizontal axis represents the percentage of DEGs among all genes of each pathway. DEGs: Differentially expressed genes. (E) The gray fan‐shaped sectors represent the top 10 most significantly enriched GO terms, the term with the highest number of enriched genes was the collagen‐containing extracellular matrix. The red dots represent highly expressed genes, and the blue dots represent genes with low expression. The inner purple fan‐shaped sections represent the *Z*‐score, with darker colors indicating more lowly expressed genes in the corresponding GO term and lighter colors indicating more highly expressed genes. (F) The expression of DEGs enriched in the collagen‐containing extracellular matrix. The red bars represent highly expressed genes, and the blue bars represent genes with low expression. The vertical axis shows the log_2_(fold change) of genes, and the horizontal axis displays the gene names. Genes marked with a purple square encode ECM components or components that inhibit ECM degradation and were mostly upregulated, whereas genes marked with a green square encode enzymes that digest the ECM and were mostly downregulated. ECM, Extracellular matrix; log_2_FC, log_2_(fold change).

A comparison of the transcriptomes of AxD and WT astrocytes revealed that a total of 1078 genes were differentially expressed, including 509 upregulated genes and 569 downregulated genes; among them, 479 genes had a log_2_(fold change) less than −1, and 406 genes had a log_2_(fold change) > 1 (Figure 5A). The genes with an absolute value of log_2_(fold change) > 1 were used for further GO and KEGG analyses. The volcano plot showed the genes whose expression was most significantly different, with more than 20 genes highlighted (Figure 5B). Among the highly expressed genes, *ACAN* encodes aggrecan, a type of chondroitin sulfate proteoglycans (CSPGs), which is a key component of the ECM in the CNS [25]. Among the genes whose expression was low, *ADAMTS8* and *MMP24* are important ECM‐degrading enzymes involved in the degradation of the ECM [26].

GO analysis of the DEGs revealed that the top 20 most significantly enriched terms were related mostly to the ECM (Figure 5C), including terms such as ECM organization (GO:0030198) and collagen‐containing ECM (GO:0062023). KEGG pathway analysis also revealed significant DEG enrichment in ECM‐related pathways (Figure 5D), including ECM‐receptor interaction (hsa04512) and cell adhesion molecules (hsa04514). Additionally, neuroactive ligand–receptor interaction (hsa04080) and the cytoskeleton in muscle cells (hsa04820) were also significantly enriched.

Furthermore, among the top 10 terms significantly enriched in the GO analysis of DEGs (Figure 5E), the term “collagen‐containing extracellular matrix” was the most significantly enriched term, with the highest number of genes. We then list all the genes enriched in the collagen‐containing ECM (Figure 5F), including some encoding ECM components, such as *ACAN* and *BCAN* [25]; some encoding proteins that inhibit ECM degradation, such as *SERPINA1*; and some encoding enzymes that degrade the ECM, such as *MMP8*, *ADAMTS5*, and *ADAMTS8* [26]. We found that most of the genes encoding ECM components, particularly CSPGs, and inhibitors of ECM degradation were highly expressed, whereas enzymes that degrade the ECM were expressed at low levels. Notably, ADAMTS family proteins were all expressed at low levels, with very large fold changes. We validated the expression levels of the key DEGs mentioned above with qPCR (Figure S3). These results indicated that in AxD, GFAP‐mutant astrocytes exhibited increased expression of ECM, which may affect myelination and neural plasticity [26], and could be one of the pathogenic mechanisms of AxD.

## 4 Discussion

In previous studies of AxD, cell and animal models have been established. AxD cellular models were mostly generated via the transfection of astrocytoma cell lines (U343MG) [27] or carcinoma cell lines (MCF7, SW13) [28]; these models cannot fully replicate AxD astrocytes. AxD animal models were developed mostly via the overexpression of GFAP through the insertion of human GFAP fragments [29, 30, 31, 32]. Few animal models with AxD‐causing mutation sites have been created [33]. Overexpression of GFAP does not adequately explain the pathogenic mechanisms of *GFAP* mutation. In contrast, patient‐derived iPSCs can differentiate directly into astrocytes while preserving the AxD mutation, which is a better platform for disease modeling and drug screening [13].

A few studies have established iPSC models of AxD [19, 34]. They found that AxD‐iPSC‐derived astrocytes exhibited abnormal organelle distribution [35], dysfunctional mitochondrial transfer [36], and inhibited the maturation and myelination of oligodendrocyte progenitor cells [6]. We are the first to focus on morphological changes and the role of the ECM in this disease by using an AxD‐iPSC model.

In this study, we generated three AxD‐iPSC lines with point mutations in *GFAP*. One of these mutation sites (p.N77S) was used to develop an iPSC line and study in vitro for the first time. We induced iPSC‐derived astrocytes through an efficient and rapid method within 21 days. The efficiency of inducing WT‐iPSC and AxD‐iPSC into astrocytes showed no statistical difference, although this does not rule out the possibility that insufficient sample size could be a contributing factor. All three AxD iPSC–derived astrocytes exhibited GFAP‐positive aggregation, resembling RFs in the AxD brain, which was not observed in WT iPSC‐derived astrocytes. We also stained astrocytes with CRYAB and vimentin, the most commonly used markers for RF, and observed positive staining within the aggregations. Electron microscopy also confirmed the deposition of RFs which aggregated along intermediate filaments. This finding confirms the previous hypothesis that mutant GFAP provides a platform that facilitates the aggregation of heat shock proteins, apoptotic proteins, and other components [22]. AxD iPSC–derived astrocytes effectively simulate the pathological phenotypes of diseases and constitute a reliable platform for further studies.

To compare WT astrocytes, we analyzed and summarized the morphological characteristics of AxD astrocytes for the first time. Based on our observations, AxD astrocytes had larger cell bodies, higher GFAP expression, and shorter but more branched and more complex processes. These morphological features are similar to those of reactive astrocytes, which are subtypes of astrocytes associated with injury, disease, or infection of the CNS [37]. The upregulation of proinflammatory factors in the brain promotes the polarization of astrocytes into reactive astrocytes, and astrocyte–microglia crosstalk also facilitates this polarization [23]. However, in AxD, changes in astrocytes are not caused by external factors. Whether the transformation of astrocytes into a reactive subtype can occur independently of external factors and whether AxD astrocytes resemble reactive astrocytes in other aspects still need to be validated through further experiments. Since GFAP forms intermediate filaments as crucial components of the cytoskeleton, we speculate that the morphological changes in AxD astrocytes are due to the pathological aggregation of GFAP, which impairs its normal cytoskeleton structure, thereby limiting the extension of astrocytic processes.

By comparing the transcriptomes of AxD astrocytes and WT astrocytes, we identified more than one thousand DEGs. GO analysis and KEGG pathway analysis revealed that these genes were most significantly enriched in the ECM. Abnormal expression of ECM‐related genes may affect the migration of astrocytes and the extension of their processes [38], which, together with cytoskeletal abnormalities, probably contribute to the morphological alterations observed in AxD astrocytes.

In the CNS, chondroitin sulfate proteoglycans (CSPGs) are important components of the ECM, including aggrecan (ACAN), brevican (BCAN), neurocan (NCAN), and versican (VCAN) [25]. Previous studies have shown that reactive astrocytes overexpress CSPGs, which inhibit axonal growth and myelination, especially in spinal cord injury models [39]. Enzymatic digestion of CSPGs or upregulating ECM‐degrading enzymes, ADAMTS and MMP, can improve nerve regeneration and myelination [40]. In Alzheimer's disease mice, the overexpression of CSPGs inhibits synapse formation in the hippocampus, whereas the enzymatic digestion of CSPGs not only rescues the loss of synaptic density but also reduces the Aβ burden [41, 42]. In conclusion, an abnormal increase in CSPGs limits axonal extension, reduces synapse formation, and inhibits myelination [26].

In this study, transcriptomic analysis indicated that the genes encoding CSPGs components were significantly upregulated, whereas the genes encoding ECM proteases were generally downregulated. The changes in ECM‐related gene expression in AxD astrocytes are very similar to those in reactive astrocytes after CNS injury. We speculate that, like the role of reactive astrocytes in CNS disease, the excessive secretion of CSPGs and the reduction in ECM proteases in AxD astrocytes may also impact the myelination and synapse formation, which play an important role in the leukodystrophy and the occurrence of epilepsy in AxD. Furthermore, since CSPGs also affect neural development, with low expression during the embryonic and early postnatal stages and gradually increasing with age [43], accompanied by a decline in neural plasticity, the overexpression of CSPGs by GFAP‐mutant astrocytes may contribute to early‐onset symptoms in AxD patients. In addition, inhibiting the abnormal expression of CSPGs may be a potential therapeutic approach for AxD. Current studies have shown that the use of competitive inhibitors of the CSPG receptor PTPσ improved myelin and axonal regeneration after spinal cord injury [44]. This target may also be applicable for the treatment of AxD.

Currently, no studies have focused on changes in the ECM in AxD. Considering the critical role of the ECM in other CNS diseases, our research provides new possibilities for identifying key molecules involved in the pathogenesis of AxD and offers new insights for exploring potential therapeutic approaches.

There are several limitations in this study. First, we observed morphological changes in AxD astrocytes, but whether these changes affect the migration and adhesion of astrocytes and the integrity of the blood–brain barrier remains to be further investigated. Second, this study highlights the significant role of the ECM in AxD, but it remains unclear which ECM component plays the most critical role, through which pathway to affects phenotype and whether intervening in the ECM can affect the prognosis of AxD. We will further explore these questions in future research.

## 5 Conclusions

We established iPSC models of AxD that retain the original GFAP mutation in AxD patients. AxD‐iPSC‐derived astrocytes successfully simulate the disease phenotype. We also identified specific morphological characteristics of AxD astrocytes that closely resemble reactive astrocytes. Additionally, transcriptomic analysis revealed that the differentially expressed genes were enriched mainly in the ECM, with a general trend toward excessive ECM secretion. This could point to a previously overlooked pathogenic mechanism in AxD and provide new possibilities for discovering new treatments.

## Conflicts of Interest

The authors declare no conflicts of interest.

## Supporting information


**Figure S1.** Astrocytes derived from AxD‐iPSC and WT‐iPSC expressed the astrocyte functional markers ALDH1L1 and EAAT2. (A) Astrocytes derived from both WT‐iPSCs and AxD‐iPSCs expressed the astrocyte marker GFAP (green), ALDH1L1 (red) and nuclear staining (Hoechst, blue); scale bars, 50 μm. (B) Astrocytes derived from both WT‐iPSCs and AxD‐iPSCs expressed the astrocyte marker GFAP (green), EAAT2 (red) and nuclear staining (Hoechst, blue); scale bars, 50 μm.
**Figure S2.** Comparison of the rapid induction protocol and traditional induction protocol. (A) Schematic diagram of the rapid induction protocol. (a–d) Light microscope images of cells on Days 3, 7, 14, and 21 during astrocyte differentiation using the rapid induction protocol. The cells gradually transition from a polygonal shape to a stellate shape. (e–l) Immunofluorescence images of cells induced using the rapid induction protocol. On Day 3, the cells express the neural stem cell marker Nestin and the astrocyte precursor cell marker CD44. By Day 7, they express astrocyte precursor cell markers CD44 and Vimentin (VIM), along with a small amount of the astrocyte marker GFAP. By Day 14, GFAP is robustly expressed, along with a small amount of S100β, and by Day 21, the majority of cells express both GFAP and S100β. (B) Schematic diagram of the traditional induction protocol, which involves three stages: neural induction, astrocyte differentiation, and astrocyte maturation. (m–o) Light microscope images of cells on Week 3 (neural induction), Week 6 (astrocyte differentiation), and Week 10 (astrocyte maturation) during astrocyte differentiation using the traditional induction protocol. The cells transition from rosettes to a stellate shape. (p–s) Immunofluorescence images of cells induced using the traditional induction protocol. At week 3, the cells express the neural stem cell markers Nestin and Sox2. By week 6, they express the astrocyte precursor cell marker CD44, and by week 10, they express astrocyte markers GFAP and S100β.
**Figure S3.** qPCR validation of key DEGs. (A–F) qPCR validation of the relative mRNA expression levels of *ACAN*, *BCAN*, *ADAMTS5*, *ADAMTS8*, *SERPINA1*, and *MMP8* (*n* = 9). The expression of *ACAN*, *BCAN*, and *SERPINA1* is upregulated in AxD astrocytes, while the expression of *MMP8*, *ADAMTS5*, and *ADAMTS8* is downregulated. The *y*‐axis values represent 2^−ΔΔ*Ct*^, with GAPDH as the reference gene. Data represent as dot plots with mean ± SD, *n* = 9 in each group with three independent experiments, one‐way ANOVA, *p < 0.05, **p < 0.01, ***p < 0.001, ****p < 0.0001; WT‐As, WT Astrocytes; AxD‐As, AxD Astrocytes.

## Data Availability

The original contributions presented in the study are included in the article and Supporting Information, further inquiries can be directed to the corresponding author.
