# Supplementary material for: Morphological Characteristics and Extracellular Matrix Abnormalities in Astrocytes Derived From iPSCs of Children With Alexander Disease
Source: CNS Neurosci Ther. 2025 Jan 27;31(1):e70240. doi: 10.1111/cns.70240 (PMC11770893; doi:10.1111/cns.70240)
Supplement: Supplementary file 1 — Figure S1. Astrocytes derived from AxD‐iPSC and WT‐iPSC expressed the astrocyte functional markers ALDH1L1 and EAAT2. (A) Astrocytes derived from both WT‐iPSCs and AxD‐iPSCs expressed the astrocyte marker GFAP (green), ALDH1L1 (red) and nuclear staining (Hoechst, blue); scale bars, 50 μm. (B) Astrocytes derived from both WT‐iPSCs and AxD‐iPSCs expressed the astrocyte marker GFAP (green), EAAT2 (red) and nuclear staining (Hoechst, blue); scale bars, 50 μm. Figure S2. Comparison of the rapid induction protocol and traditional induction protocol. (A) Schematic diagram of the rapid induction protocol. (a–d) Light microscope images of cells on Days 3, 7, 14, and 21 during astrocyte differentiation using the rapid induction protocol. The cells gradually transition from a polygonal shape to a stellate shape. (e–l) Immunofluorescence images of cells induced using the rapid induction protocol. On Day 3, the cells express the neural stem cell marker Nestin and the astrocyte precursor cell marker CD44. By Day 7, they express astrocyte precursor cell markers CD44 and Vimentin (VIM), along with a small amount of the astrocyte marker GFAP. By Day 14, GFAP is robustly expressed, along with a small amount of S100β, and by Day 21, the majority of cells express both GFAP and S100β. (B) Schematic diagram of the traditional induction protocol, which involves three stages: neural induction, astrocyte differentiation, and astrocyte maturation. (m–o) Light microscope images of cells on Week 3 (neural induction), Week 6 (astrocyte differentiation), and Week 10 (astrocyte maturation) during astrocyte differentiation using the traditional induction protocol. The cells transition from rosettes to a stellate shape. (p–s) Immunofluorescence images of cells induced using the traditional induction protocol. At week 3, the cells express the neural stem cell markers Nestin and Sox2. By week 6, they express the astrocyte precursor cell marker CD44, and by week 10, they express astrocyte markers GFAP [file CNS-31-e70240-s001.docx]

**
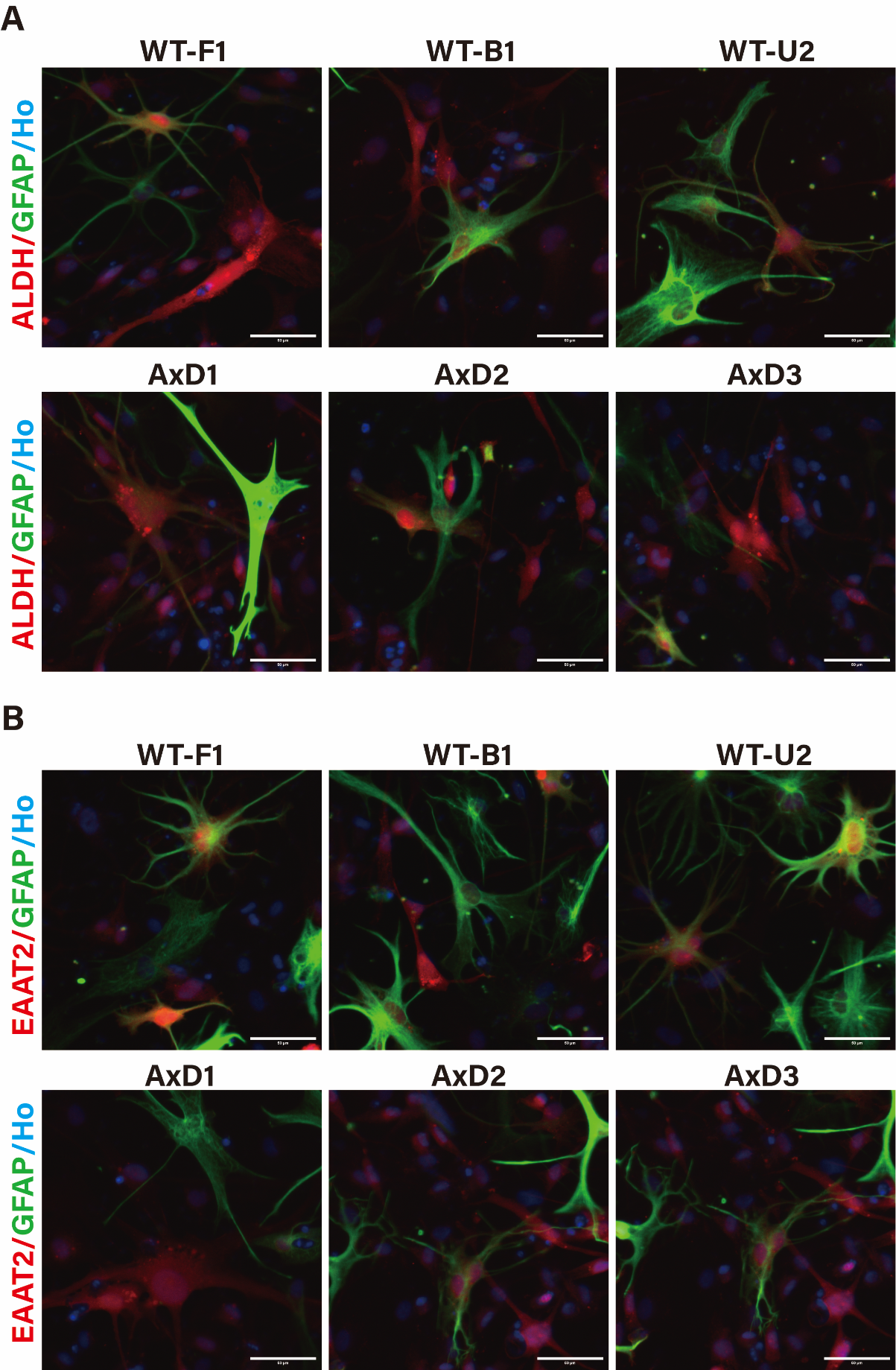
Supplementary Figure 1 Astrocytes derived from AxD-iPSC and WT-iPSC expressed the astrocyte functional markers ALDH1L1 and EAAT2. A**. Astrocytes derived from both WT-iPSCs and AxD-iPSCs expressed the astrocyte marker GFAP (green), ALDH1L1(red) and nuclear staining (Hoechst, blue); scale bars, 50 μm. **B**. Astrocytes derived from both WT-iPSCs and AxD-iPSCs expressed the astrocyte marker GFAP (green), EAAT2(red) and nuclear staining (Hoechst, blue); scale bars, 50 μm.


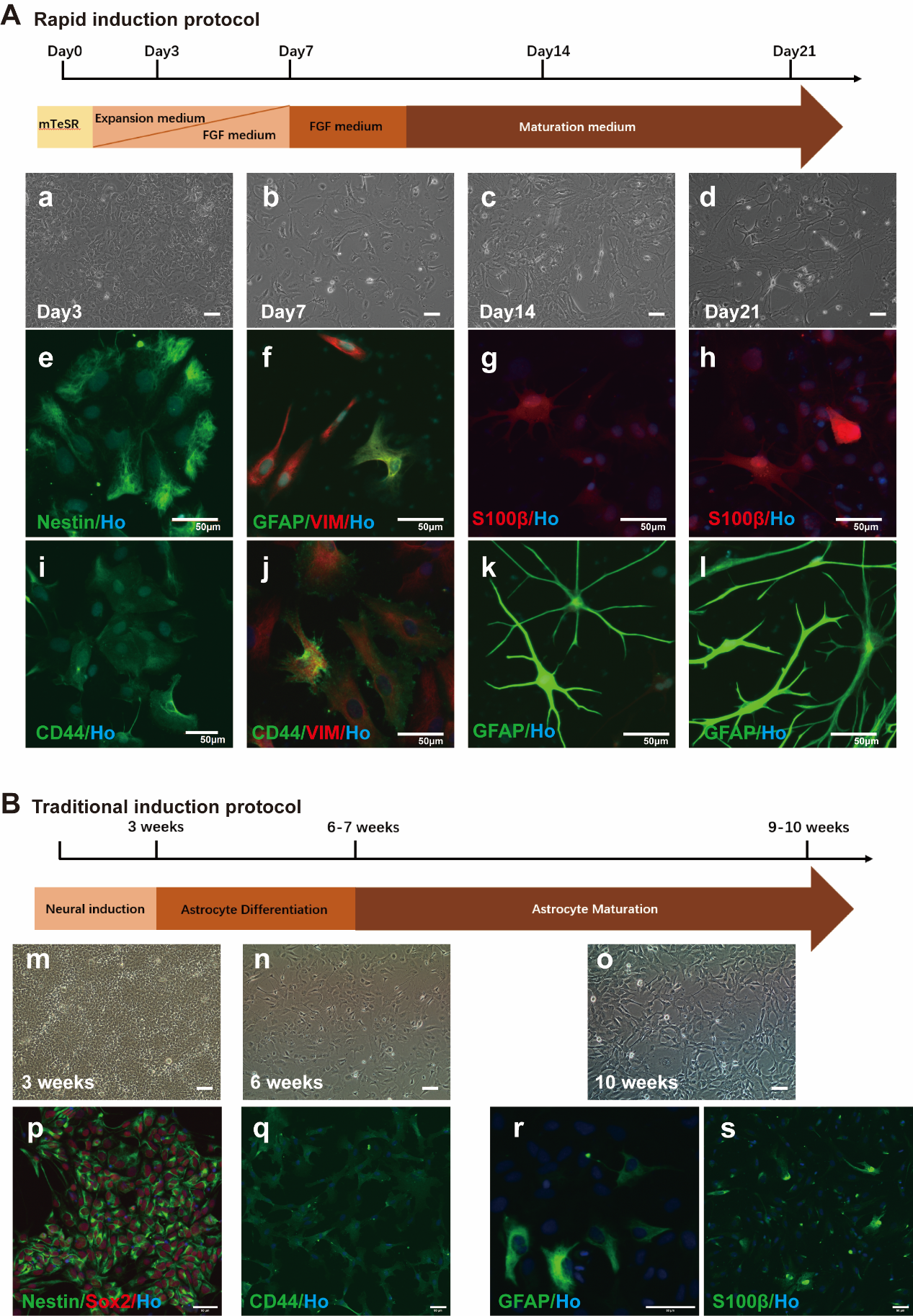
**Supplementary Figure 2 Comparison of the rapid induction protocol and traditional induction protocol. A.** Schematic diagram of the rapid induction protocol. **a-d**. Light microscope images of cells on days 3, 7, 14, and 21 during astrocyte differentiation using the rapid induction protocol. The cells gradually transition from a polygonal shape to a stellate shape. **e-l**. Immunofluorescence images of cells induced using the rapid induction protocol. On day 3, the cells express the neural stem cell marker Nestin and the astrocyte precursor cell marker CD44. By day 7, they express astrocyte precursor cell markers CD44 and Vimentin (VIM), along with a small amount of the astrocyte marker GFAP. By day 14, GFAP is robustly expressed, along with a small amount of S100β, and by day 21, the majority of cells express both GFAP and S100β. **B**. Schematic diagram of the traditional induction protocol, which involves three stages: neural induction, astrocyte differentiation, and astrocyte maturation. **m-o**. Light microscope images of cells on week 3 (neural induction), week 6 (astrocyte differentiation), and week 10 (astrocyte maturation) during astrocyte differentiation using the traditional induction protocol. The cells transition from rosettes to a stellate shape. **p-s**. Immunofluorescence images of cells induced using the traditional induction protocol. At week 3, the cells express the neural stem cell markers Nestin and Sox2. By week 6, they express the astrocyte precursor cell marker CD44, and by week 10, they express astrocyte markers GFAP and S100β.


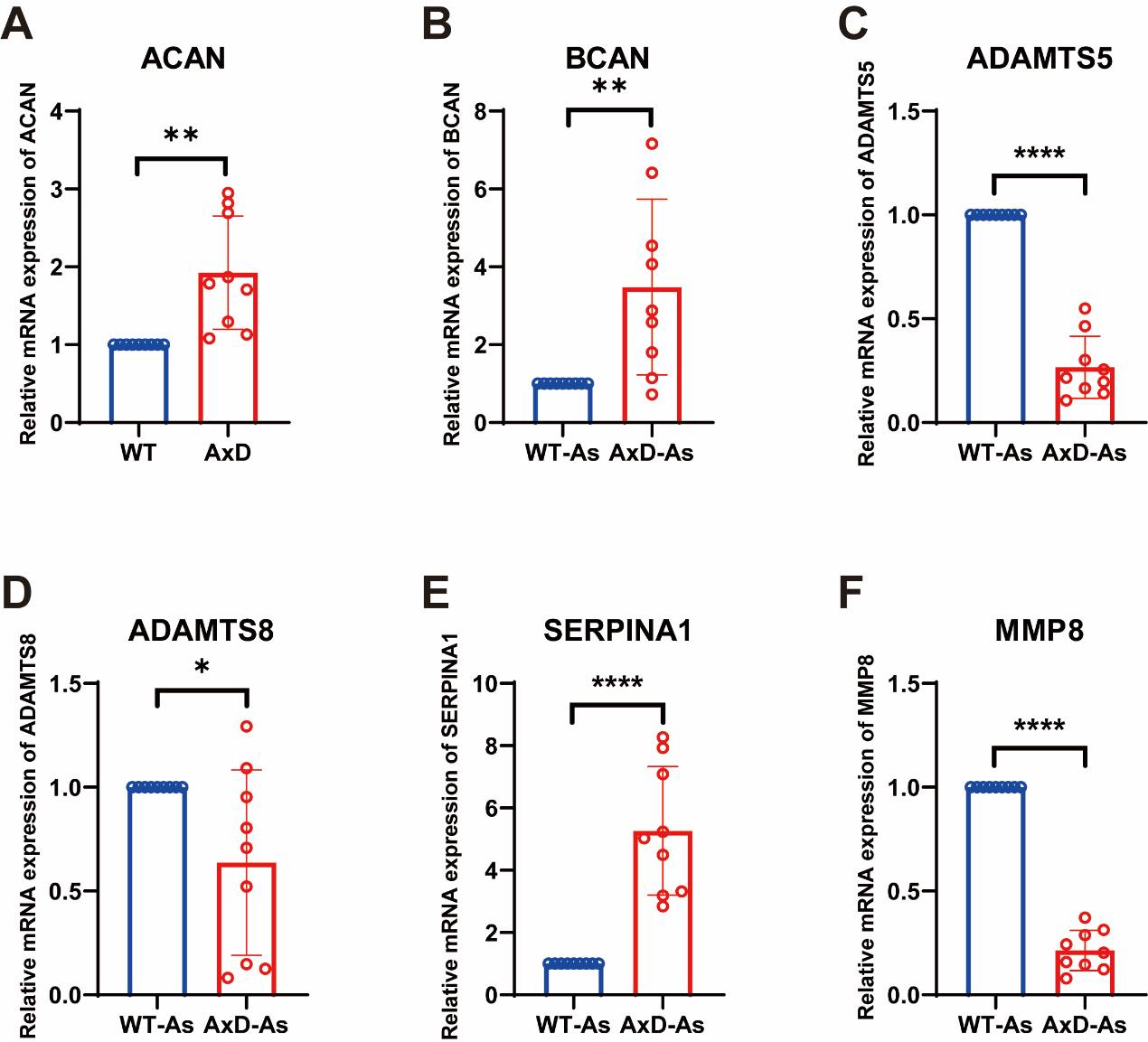


**Supplementary Figure 3 qPCR validation of key DEGs. A-F**: qPCR validation of the relative mRNA expression levels of *ACAN, BCAN, ADAMTS5, ADAMTS8, SERPINA1*, and *MMP8* (n=9). The expression of *ACAN, BCAN,* and *SERPINA1* is upregulated in AxD astrocytes, while the expression of *MMP8, ADAMTS5,* and *ADAMTS8* is downregulated. The y-axis values represent 2^-ΔΔCT, with GAPDH as the reference gene. Data represent as dot plots with mean ± SD, n = 9 in each group with three independent experiments, one‐way ANOVA, *: p< 0.05, **: p< 0.01, ***: p < 0.001, ****: p < 0.0001, WT-As: WT Astrocytes, AxD-As: AxD Astrocytes
